# Making the Cut: Implementing a Low Cost, Low Fidelity Simulation Model for Teaching Emergency Thoracotomy Procedure

**DOI:** 10.7759/cureus.8088

**Published:** 2020-05-13

**Authors:** Alanna O'Connell, Xiao Chi Zhang, Megan Crossman, Sarah Misuro, Megan Stobart-Gallagher

**Affiliations:** 1 Emergency Medicine, Thomas Jefferson University, Philadelphia, USA; 2 Emergency Medicine, Thomas Jefferson University Hospital, Philadelphia, USA; 3 Emergency Medicine, Einstein Medical Center Philadelphia, Philadelphia, USA

**Keywords:** simulation trainer, thoracotomy, medical education, simulation in medical education, procedure training, trauma, low fidelity

## Abstract

Emergent thoracotomy is a rare but high-stakes procedure for trauma patients. Emergency medicine residents are expected to perform this procedure after graduation, but few get the opportunity to perform it, leading to suboptimal performance and patient morbidity and mortality. Previous low-cost thoracotomy trainers revolve around modifying an existing costly thoracotomy task trainer or bear limited resemblance to actual landmarks and anatomy. This study attempts to bridge this gap by creating a low-cost model with supplies found at most home improvement/craft stores that is more anatomically accurate. We constructed a low fidelity model, which residents ultimately found to be helpful in mastering this rare procedure, and after the training session, they reported a greater level of comfort and familiarization with the procedure.

## Introduction

Emergency medicine (EM) residencies train resident physicians to perform, practice, and eventually, master common emergent procedures necessary to temporize or resolve medical emergencies. However, there are many procedures that the emergency medicine resident is expected to be knowledgeable of, but are very infrequently done. These high acuity low occurrence (HALO) procedures must be performed in high stake occurrences on some of the sickest patients, prompting further addition to mastering these skill-sets during residency training. Given how infrequently they are done, many residents feel uncomfortable performing them. Simulation has become a method for training residents on how to perform HALO procedures using innovative, high fidelity, and low-cost task trainers (specialized models designed to teach specific skill), however, there is a dearth of high-fidelity task trainers to instruct EM residents on how to perform one of the most HALO trauma procedures - emergent thoracotomy [1].

We sought to construct a low-cost, low fidelity model that sought to bridge that gap in knowledge and experience with our residents. Commercially available models were available but found to be cost-prohibitive [2, 3]. Previous low-cost thoracotomy trainers have been proposed in the past but typically revolve around modifying a costly existing simulation model or are very low fidelity and bear limited resemblance to actual landmarks and anatomy [4,5].

We propose that our low-cost, reusable task thoracotomy trainer model: 1) closely resembles real chest anatomy, 2) enables EM residents to practice the steps of the procedure, and 3) allows learners to identify the indications, steps, and safety measurements for an ED thoracotomy.

## Technical report

Design considerations and education context

Senior post-graduate year (PGY) 2 and PGY3 residents at a level one trauma center/tertiary care academic center were enrolled as part of their structured weekly didactic. This was designed for a single session lasting approximately 90 minutes of their mandatory half-day didactic time. Students were provided with a brief background lecture; then, residents took turns in groups of two performing the procedure using the model under the guidance of an instructor. After attempting the procedure, they were asked to complete a voluntary short survey intended to gauge their level of comfort with the procedure after this course.

Design protocol and development

All items required to create this trainer were purchased by the principal investigator and are readily available and inexpensive at most home improvement/craft stores. These include 3x1 hardwood board, electrical wire, red felt (to cover base), screwdriver, screws, craft wire, white fabric, craft foam (with and without backing), clay, paint, pleather, zippers, plastic wrap (red-colored works best), plastic sandwich bags.

Step 1

**Figure 1:**
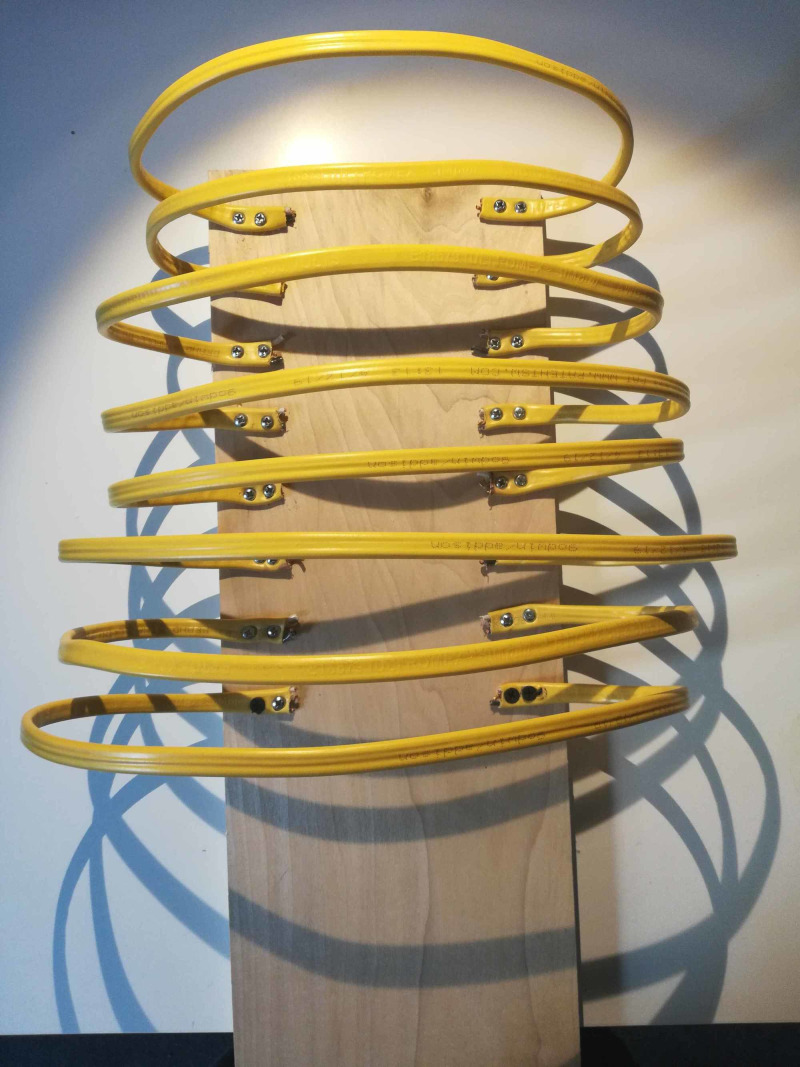
Baseboard with electric wire attached

A piece of scrap wood was selected to be used as a backboard for the model. A flat wide electrical wire was purchased at a home improvement store and cut into small medium and large strips. These were affixed to the backboard using two screws to each side for stability with the smallest strips at the top and larger towards the bottom to create a similar shape to ribs, as indicated in Figure 1.

Step 2

**Figure 2:**
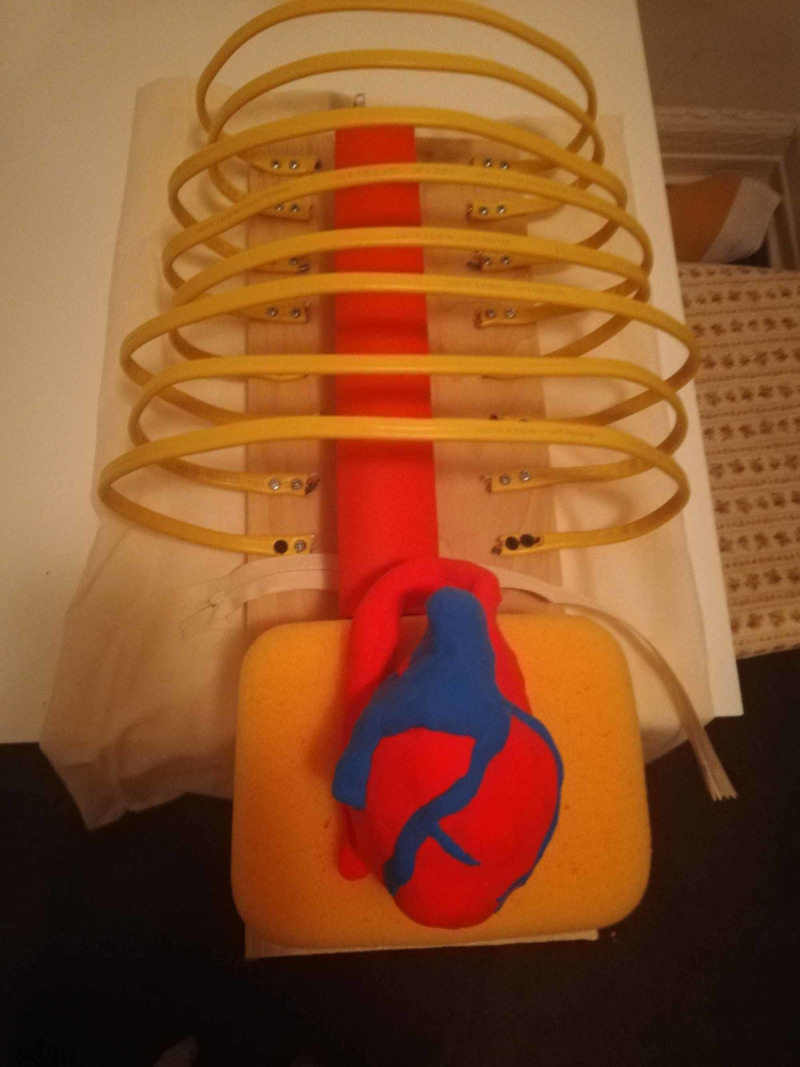
Completed esophagus and aorta in the heart model

Several different types of the heart were trialed, but what seemed to work best was a rudimentary sculpture made of soft air-dry clay, available at most craft stores. This allowed for both accurate sizing and kept the model from being too heavy. An esophagus was made of simple red craft foam rolled into a tube and glued into shape. An aorta was made of the red craft foam that came with backing attached (backing left on foam) in a similar manner. This made the aorta less pliable than the esophagus. See Figure 2.

Step 3

The wire was threaded down the middle of the ribs to create a sternum and give the ribs more stability while remaining pliable. The esophagus and aorta were screwed into place on the backboard, and the heart was attached via elastic band to the backboard so it could be "delivered" during the procedure. Optional is to cover ribs and sternum with white fabric, and the wood back board with red felt to give it a more realistic look.

Step 4

**Figure 3:**
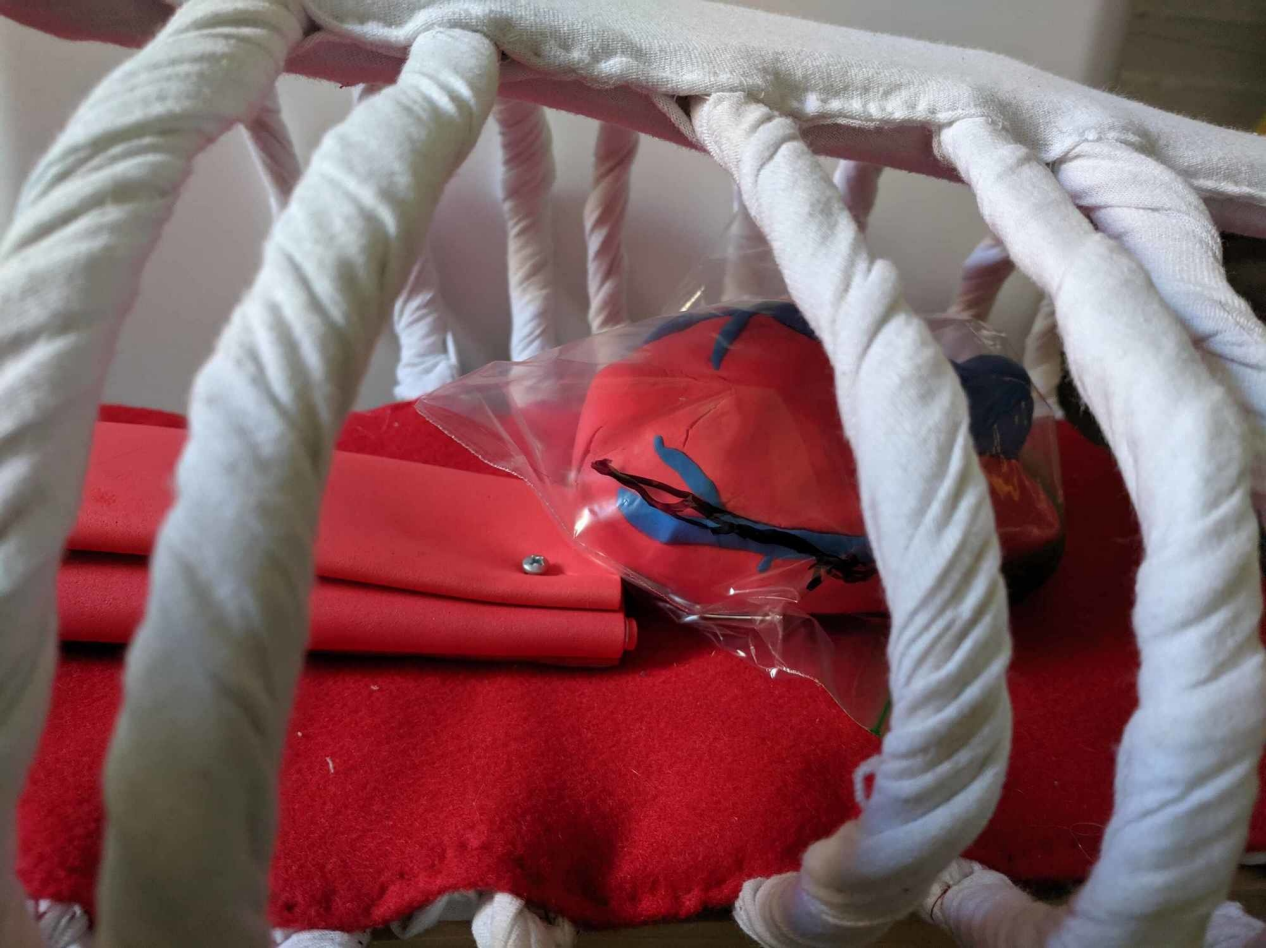
Heart encased in plastic bag pericardium

Plastic sandwich bags were used for the pericardial sac as they are cheap and easily replaceable. A vagus nerve was drawn on with a marker. A more realistic approach could be to glue on a piece of rubber band for tactile feedback; however, this would need a lot more preparation. The plastic bag was placed around the heart and partially zippered to secure (see Figure 3).

Step 5

**Figure 4:**
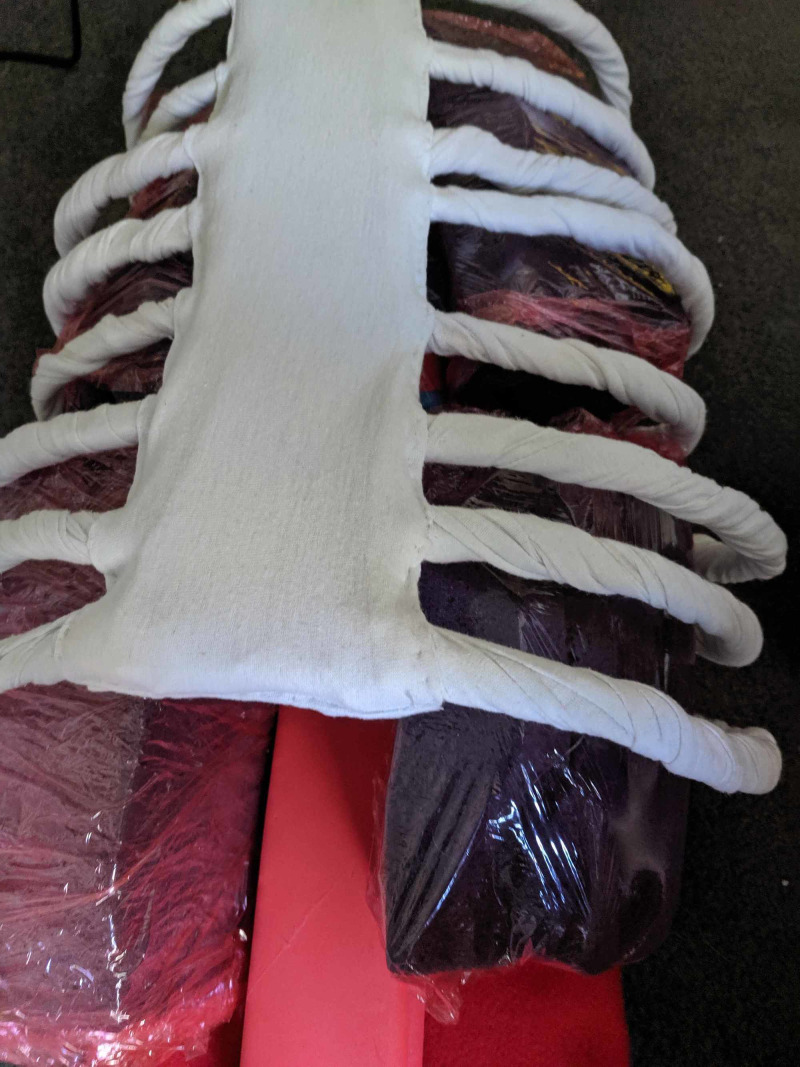
Lungs created from painters sponges dyed purple covered in red plastic wrap

Lungs were constructed of painters sponges that were cut to shape then dyed purple with fabric paint. These were slid into place (not secured) and were easily removable if needed to make adjustments between learners. These could optionally be covered with red plastic wrap as "pleura" for a more realistic appearance/feel (see Figure 4).

Step 6

**Figure 5:**
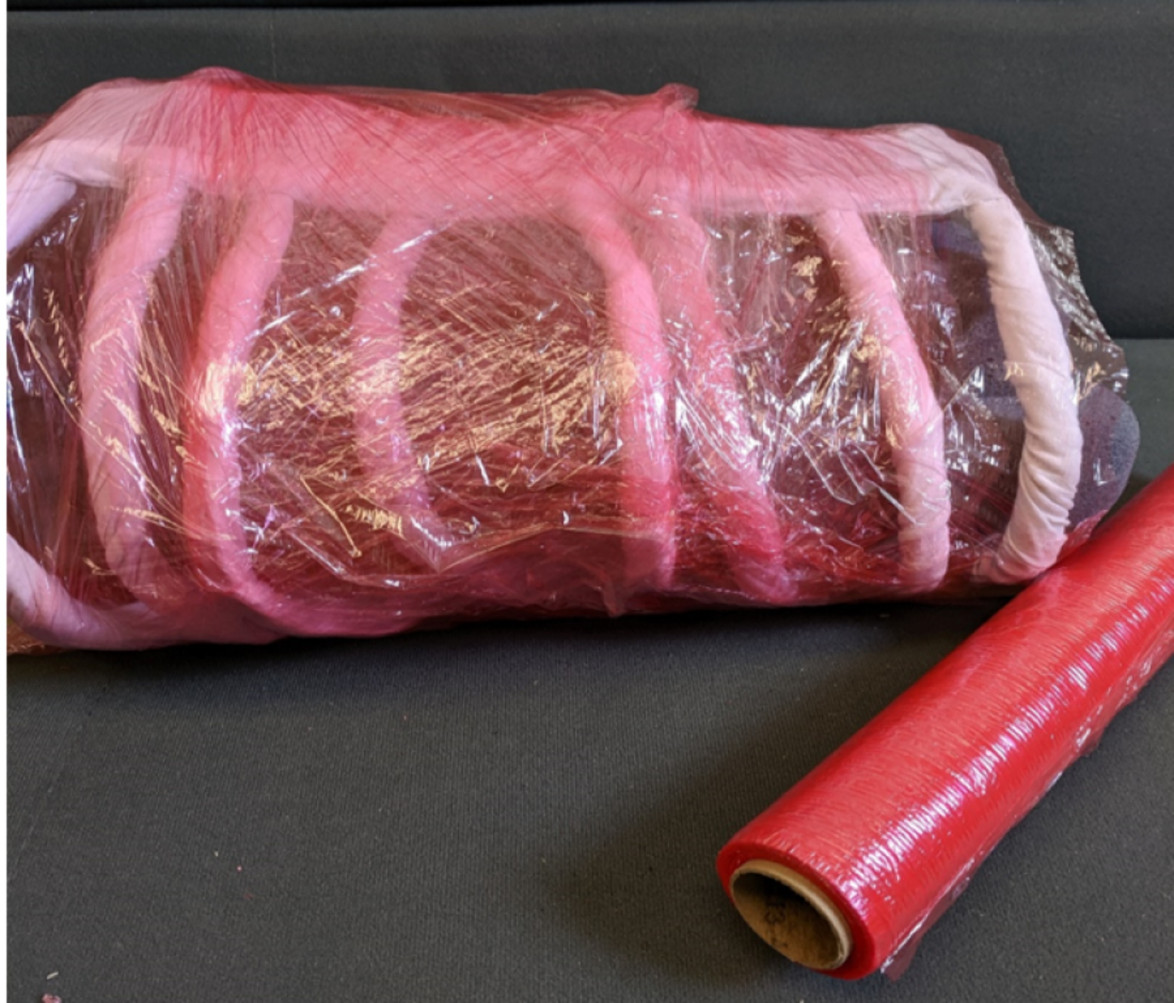
Model wrapped in red plastic wrap to create intercostal muscles

The model was then wrapped in red plastic wrap to create intercostal muscles. This again could be quickly replaced between learner attempts and can be cut during the procedure similar to how the intercostals would be (see Figure 5).

Step 7

**Figure 6:**
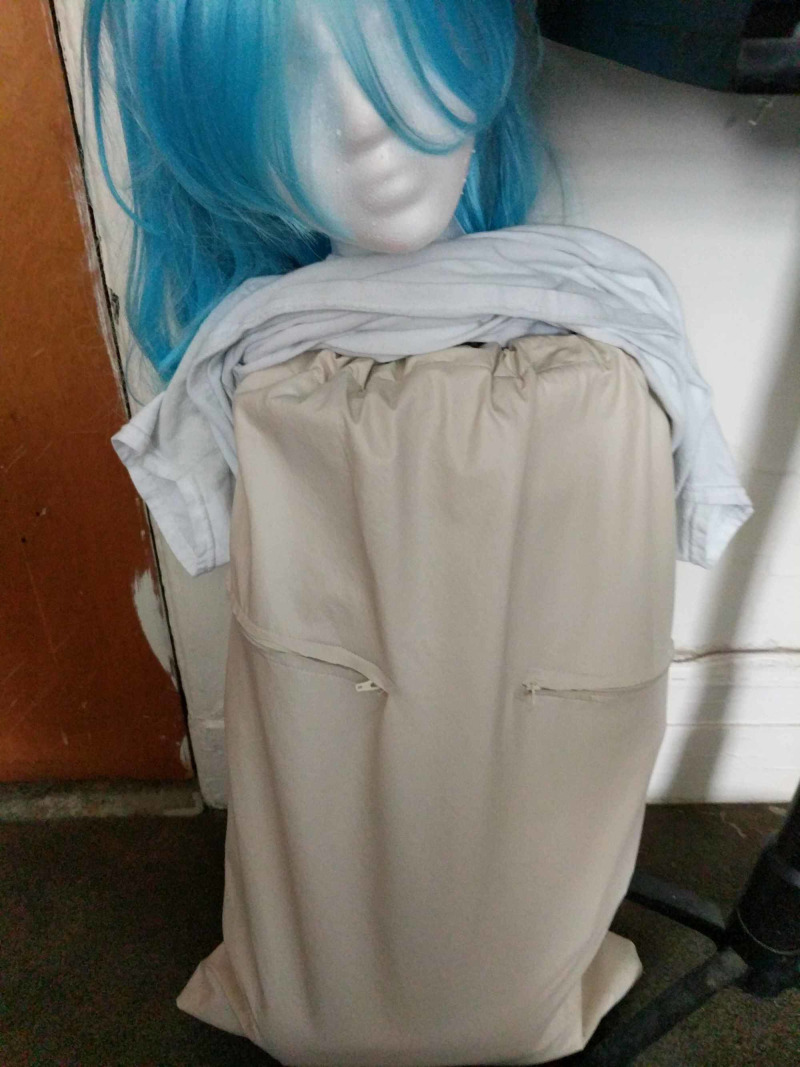
Vinyl fabric "skin" covering the model with zippers at incision sites

The skin was created using vinyl fabric. This was sewn into a bag to cover the model with a drawstring top and a zipper down the back for quick removal to replace the pericardial sac, reshape ribs, and rewrap intercostal muscles between learners. This approach was created to save on preparation time (rather than creating 30+ skins to cut) and maximize the re-usability of the model. Zippers were sewn into the incision sites to simulate the incisions placed. See Figure 6.

Step 8

**Figure 7:**
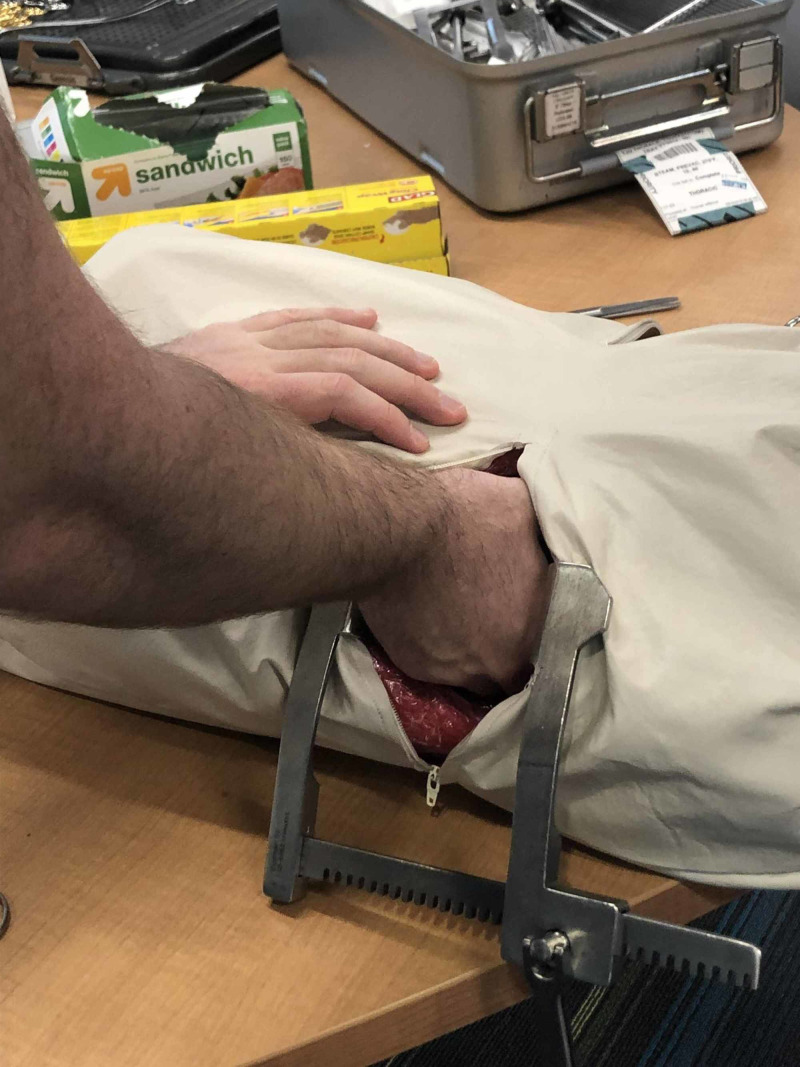
Model in use during classroom session

The model was then used in the classroom session. Between learners, the instructor should only have to quickly replace pericardial sack and rewrap with plastic wrap. The average time to reset between learner attempts was less than a minute to reset. See Figure 7.

Evaluation methods

A post-session survey was given to residents using Brookfield's Critical Incident Questionnaire, where the residents were first queried their year of training and prior experience with the ED thoracotomy [6]. They then responded to questions regarding their comfort with the procedure after the session, if they thought the model was helpful for learning, and if they felt it was anatomically accurate to the procedure. These questions were answered on a Likert scale from strongly disagree → neutral → agree → strongly agree. They then filled out the Brookfield Critical Incident Questionnaire to help assess what was helpful and what could be improved for this session. 

Results

**Figure 8:**
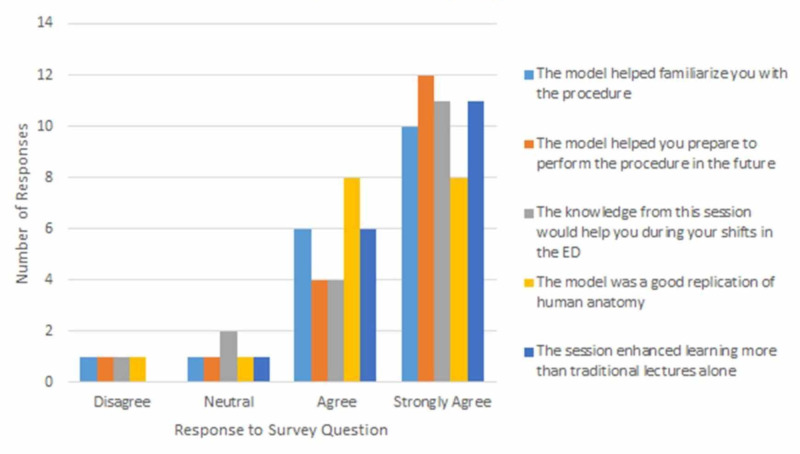
Resident survey responses

Of the approximate 30 participants in the session, 18 returned their surveys completed. In this survey, 16% reported they had performed this procedure before. Seventy-seven percent of the participants had seen a thoracotomy but never performed the procedure. A small number of residents reported having performed (or assisted) the procedure in the past (16%), which is consistent with what would be expected in this type of high acuity, low occurrence procedure. Over 66% of surveyed senior residents completed the didactic session feeling better prepared to perform the procedure in real-time in the future. The majority of the residents on the Brookfield Critical Incident Questionnaire responded positively to the activity with mostly "agree" or "strongly agree" responses. The responses are summarized in Figure 8.

## Discussion

Overall, the survey responses to questions about the efficacy of this task trainer were positive with a small cohort of neutral or negative responses.

According to the Brookfield Critical Incident Questionnaire, most residents seemed to think this activity was helpful and felt engaged for most of the session. They felt less engaged when they were waiting for their turn to attempt the procedure. The majority of the residents agreed or strongly agreed that the model helped familiarize them with the procedure, helped prepare them to perform the procedure in the future, that this would help them during their shifts in the ER, that the model was a good replication of human anatomy, and that this session enhanced learning more than traditional lectures alone.

This study was limited to a small group at a single center. Further assessment of its utility among larger groups at various institutions is needed. However, based on our data, the trainer can be a helpful adjunct to training EM residents on how to perform this relatively rare procedure in the ED with low cost to the program. It was also very easy and fast to reset between learners. In the Brookfield Critical Incident Questionnaire, residents mostly reported feeling disengaged when waiting for their turn to practice. For future sessions, more than one model/instructor may be helpful for increasing the resident’s ability to practice this procedure and be engaged in learning.

## Conclusions

The residents felt that the model was helpful in mastering this HALO procedure and after the training session they reported a greater level of comfort and familiarization with the procedure. The model itself should be easy to make and far cheaper than commercially available models to assist with training residents. For future study and sessions more than one model could be made to allow additional time for residents to practice. Future models could also be designed so that a true clam-shell thoracotomy (right and left side incisions with cutting of sternum) could be practiced.
